# 30 Years of Trajectory: Many Challenges but Also Many Reasons to Celebrate

**DOI:** 10.5935/1678-9741.20160018

**Published:** 2016

**Authors:** Domingo M. Braile

**Affiliations:** 1 BJCVS

This historical issue kicks off celebrations after 30 years of continuous publication of the Revista Brasileira de Cirurgia Cardiovascular, known today as the Brazilian Journal of Cardiovascular Surgery (BJCVS).

These 30 years represent the history of our Society.

The journal was founded by idealists led by Dr. Adib Jatene, and they represent the pioneers of cardiac surgery in Brazil and South America.

Professor Adib was the Editor-in-Chief for the first 10 years, from 1986 to 1996.

Our current President followed in his footsteps, showing his permanent dedication to the Brazilian Society of Cardiovascular Surgery (BSCVS).

Prof. Fabio Jatene was the Editor-in-Chief during 6 years, from 1996 to 2002.

In 2002, I put myself at disposal and I have been the Editor-in-chief since then.

Due to our growth and complexity, our Associated Editors currently play a bigger role, in which they are responsible for the entire process of dealing with the articles they are attributed. The Editor-in-Chief still has the final word.

Space limitations do not allow me to name every single person that has worked, without pay or advantage, for the greater good of the BJCVS.

I ask all the coleagues to access at the journal's webpage at www.bjcvs.org, in the "Editorial Board" session to see the names of those who throughout our history have gone to great lengths to create and maintain the BJCVS and that is why they deserve to be praised by all.

In addition, at the end of every issue, all our reviewers are listed, to whom I pay tribute for sacrificing their leisure time not only to review the manuscripts, but especially to guide the authors who show potential, helping them conform to scientific methods, to the guidelines of the journal and standard language, thereby acting as professors.

This kind of attitude has gradually improved the level of our publications, though we still have to improve our work.

Changing the name of the journal on its 30^th^ anniversary was a difficult decision, but a necessary one in order to meet the needs of a new international reality.

Nowadays, English is the lingua franca of scientific communication.

We could not be tied down by nostalgia, remaining unread and not understood by the international community.

You are witness to the care we took to make this transition.

First, as required by SciELO, PubMed etc., the abstract was published in Portuguese and English.

Then, for a long time, we became a bilingual publication, which was very interesting, but also a demanding job since for every issue we actually published two "journals", one in Portuguese and the other in English.

The titles of the articles continued in Portuguese for some time, until we decided to publish them in English to call the attention of readers who did not understand our language.

We also modified our submission system, which became fully in English to make it easier for researchers from other countries.

Such measures had some effect as we have received articles from Portugal, Colombia, United States, Canada, Germany, Iraq, Iran, Mexico (where we are in a Portal similar to SciELO), and many from China and Turkey, but not enough to increase our citations and Impact Factor, which is now our main goal.

We are in almost all international databases, allowing easy access to our articles, which is essential to attract readers, especially because our journal is completely free, both for authors and readers.

The change in our designation to English required several démarches, both in Brazil and abroad.

Finally, it became a reality, primarily because we had had the designation in English as a subtitle for a long time!

Another question arose: whether to completely change our identity and remove the word "Brazilian", following in the footsteps of many other publications that do not identify their country of origin.

Many people helped us with this task, Professor Abel Packer, Director of SciELO, being one of them, who insisted we should not lose our national identity.

I believe it was a wise decision. Brazil is much greater than the crises it faces. It is essential to show what we are capable of in order to stand amidst other nations.

Among the advantages of producing our issues in only one language, there was an important byproduct: we went from quarterly to bimonthly publication, that is, six issues per year.

I would like to call your attention to an important fact: every economic sacrifice has been made, but the new changes will have been in vain unless we publish work of proven scientific value.

Dear colleagues, I ask you to ponder these terms. We have a modern tool in accordance with global standards; however, we will not fall within the scope of leading publications if our articles are mediocre.

Our constant obsession with having a better impact factor and, as a result, a Qualis ranking compatible with our specialty, will suffer another drawback: due to the change of name, the BJCVS citation will be only in English and thereby counted by Thomson and Scopus from now on. These are problems to be faced in order to improve for the future.

## ARTICLES

In this issue, there are 13 articles, six originals, three reviews, one clinical-surgical correlation, one brief communication, one experimental work, and a letter to the editor.

Among these articles, we have four international studies - Transposition of Great Arteries with Intramural Coronary Artery: Experience with a Modified Surgical Technique (page 15), from India; Comparison of Transcatheter Aortic Valve Implantation *versus* Surgical Aortic Valve Replacement to Improve Quality of Life in Patients >70 Years of Age with Severe Aortic Stenosis (page 1), from Turkey; and Aberrant Origin of Vertebral Artery and its Clinical Implications (page 52) and Myocardial Bridging (page 60), from China.

## CME

In this BJCVS issue, the following articles are available to test the Continuing Medical Education (CME): "Results of Open and Endovascular Abdominal Aortic Aneurysm Repair According to the E-PASS Score" (page 22); "Congenital Heart Disease and Impacts on Child Development" (page 31); and "Double Aortic Arch Associated with Pulmonary Atresia with Ventricular Septal Defect" (page 63)

## BJCVS 30 YEARS

It is with great joy that we also publish reviews celebrating our 30^th^ anniversary, written with warmth by the following colleagues:

Marcela da Cunha Sales, "The Evolution of Cardiac Surgery in Brazil and the BJCVS" (page 74); Vinicius José da Silva Nina, "The 30^th^ Anniversary of the Brazilian Journal of Cardiovascular Surgery" (page 75); Rubens Tofano de Barros, "BJCVS: A Success Story" (page 77); Milton de Miranda Santoro, "The Myth of Atlas" (page 78); and Luiz César Guarita-Souza, "The Thirty Years of the Brazilian Journal of Cardiovascular Surgery" (page 79).

In the next issues, we wish to have many more descriptions of the history of the Brazilian cardiovascular surgery, including the BJCVS.

## ACKNOWLEDGEMENT

Among all the changes we have been through, there is one more: the resignation of Ricardo Brandau Quitete, who throughout the last 13 years helped us shape the profile of the journal. That is how life is, things change and we have to change along. All BSCVS partners and I can only thank Brandau for his dedication and work and wish him success in the new challenges to come.

## 43^rd^ BRAZILIAN CONGRESS OF CARDIOVASCULAR SURGERY

From April 7 to 9, the 43^rd^ Brazilian Congress of Cardiovascular Surgery will take place at Centro de Eventos do Ceará in Fortaleza, CE.

Besides the scientific program, containing innovations that make it an all-around event, on par with similar international events, this year the Congress will mark the celebration of the 30^th^ anniversary of the BJCVS.

This year's theme is "Innovation, New Horizons in Cardiovascular Surgery".

At the same time, the 6^th^ Symposium of Nursing in Cardiovascular Surgery, the 6^th^ Symposium of Physiotherapy in Cardiovascular Surgery, the 5^th^ Academic Congress in Cardiovascular Surgery, the 34^th^ Brazilian Congress of Cardiopulmonary Bypass, the Female Surgeons Meeting, and the Residents Meeting will take place.

With the purpose of discussing the most recent advances in surgical techniques and the incorporation of new technology, we will have important international guests.

The presence of several international guests consolidates the growth and fundamental integration of BSCVS with international Societies and values the essential exchange of knowledge. In addition, participants will enjoy moments with colleagues and family as well as leisure time visiting the beautiful capital of Ceará.

I congratulate the Board of the BSCVS and the Organizing Committee, coordinated by Dr. Glauco Lobo, on the effort made so that the scientific program was interesting for all the Congress participants.

On April 8^th^, from 10am to 12pm in Auditorium 7, there will be a meeting of the Editorial Board of the BJCVS with Associate Editors and Editorial Board members, also open to all members. We will address very strongly the issues that I mentioned above and it will be of great importance to have all suggestions and criticisms in this transitional phase, so that we can improve the journal and make it in accordance with the requirements of all Databases and aspiration of our readers around the globe.

Warm regards,

Domingo M. Braile Editor-in-Chief - BJCVS

